# Aquaporin-11 (AQP11) Expression in the Mouse Brain

**DOI:** 10.3390/ijms17060861

**Published:** 2016-06-01

**Authors:** Shin Koike, Yasuko Tanaka, Toshiyuki Matsuzaki, Yoshiyuki Morishita, Kenichi Ishibashi

**Affiliations:** 1 Division of Pathophysiology, Meiji Pharmaceutical University, 2-522-1 Noshio, Kiyose, Tokyo 204-8588, Japan; ystanaka@my-pharm.ac.jp (Y.T.); kishiba@my-pharm.ac.jp (K.I.); 2 Division of Analytical Biochemistry, Meiji Pharmaceutical University, 2-522-1 Noshio, Kiyose, Tokyo 204-8588, Japan; 3 Department of Anatomy and Cell Biology, Gunma University Graduate School of Medicine, Maebashi, Gunma 371-8511, Japan; matoshi@gunma-u.ac.jp; 4 Division of Nephrology, Saitama Medical Center, Jichi Medical University, 1-847 Ohmiya, Saitama-City, Saitama 330-8503, Japan; ymori@jichi.ac.jp

**Keywords:** blood brain barrier, brain edema, AQP4, choroid plexus, pia mater

## Abstract

Aquaporin-11 (AQP11) is an intracellular aquaporin expressed in various tissues, including brain tissues in mammals. While AQP11-deficient mice have developed fatal polycystic kidneys at one month old, the role of AQP11 in the brain was not well appreciated. In this study, we examined the AQP11 expression in the mouse brain and the brain phenotype of AQP11-deficient mice. AQP11 messenger ribonucleic acid (mRNA) and protein were expressed in the brain, but much less than in the thymus and kidney. Immunostaining showed that AQP11 was localized at the epithelium of the choroid plexus and at the endothelium of the brain capillary, suggesting that AQP11 may be involved in water transport at the choroid plexus and blood-brain barrier (BBB) in the brain. The expression of AQP4, another brain AQP expressed at the BBB, was decreased by half in AQP11-deficient mice, thereby suggesting the presence of the interaction between AQP11 and AQP4. The brain of AQP11-deficient mice, however, did not show any morphological abnormalities and the function of the BBB was intact. Our findings provide a novel insight into a water transport mechanism mediated by AQPs in the brain, which may lead to a new therapy for brain edema.

## 1. Introduction

Aquaporins (AQPs) are a family of transmembrane proteins transporting water and small solutes (reviewed in [1,2,3,4]). AQPs have six membrane-spanning α-helices and a pair of highly conserved NPA (asparagine–proline–alanine) motifs forming a pore [5]. There are 13 members of AQPs in mammals (AQP0-12), which have specific tissue expression patterns to regulate water and/or solute transports in various organs. However, their physiological and pathological importance has not yet been fully clarified even after the analysis of AQP deficient mice and humans [6].

The AQPs in mammals are divided into three subfamilies on the basis of primary structures and functions [1,2,3,4]. Each has a signature sequence for classification as detailed in [1]. The first subfamily, a classical AQP (CAQP), is a water-selective AQP, such as AQP1. The second subfamily, an aquaglyceroporin (AQGP), transports glycerol as well as water, such as AQP3. The third subfamily is a superaquaporin (SAQP) whose NPA boxes are distantly related to other AQPs. This subfamily includes AQP11 and AQP12, which are localized inside the cell, thereby making their functional studies difficult [7,8].

AQP11 is expressed highly in the testis and thymus, and moderately in the kidney, liver, and intestine [7]. In the kidney, AQP11 is expressed in the proximal tubule and its disruption produced fatal dilated and cyst-like proximal tubules within a month after birth [7]. Such development tubular defects may be caused by an abnormal glycosylation of polycystin-1 (PC-1), a responsible gene product of polycystic kidney disease 1 (PKD1) for human autosomal polycystic kidney disease [9].

As most AQPs play a role in edema formation and osmoregulation in human, they may also be important in the formation of brain edema [10]. In fact, AQP4 has been shown to modulate water transport at the blood-brain barrier (BBB) to aggravate brain edema after brain infarction [11] but also to facilitate the recovery from vasogenic brain edema [12], although its absence does not change BBB morphology [13]. Another AQP expressed in the brain, AQP1, has been shown to facilitate cerebrospinal fluid production in the choroid plexus [14].

Although AQP11 is expressed in the brain, its localization and role in the brain have not yet been well appreciated. One study has reported the AQP11 localization at neurons in the rat brain [15]. However, further studies have not yet been done since then.

The purpose of this study was to localize AQP11 in the mouse brain to correlate with a neural phenotype of AQP11-deficient mice, which will give an insight into the role of AQP11 in the brain.

## 2. Results

### 2.1. Expression of AQP11 mRNA and Protein in the Mouse Brain

**Figure 1:**
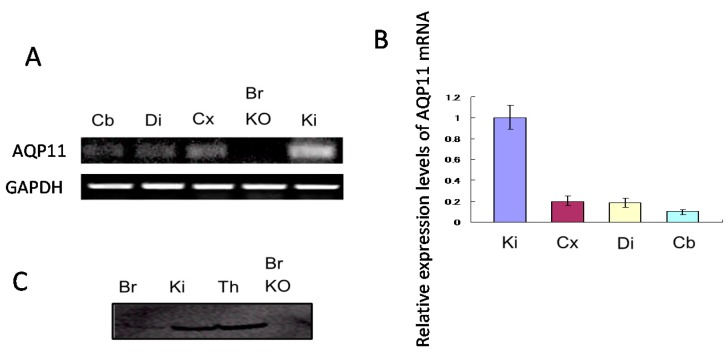
Expression levels of Aquaporin-11 (AQP11) in the brain (**A**) Reverse transcription (RT)-PCR for AQP11 messenger ribonucleic acid (mRNA) in wild type P28 mouse brain and kidney of AQP11-deficient mouse brain; (**B**) Relative abundance of brain AQP11 mRNA in wild-type mouse was compared with that of the kidney. All signals were normalized to the glyceraldehyde-3-phosphate dehydrogenase (GAPDH) mRNA signal. Each bar represents mean ± SD of three experiments; (**C**) Immunoblotting for AQP11 of P28 in the brain, thymus, and kidney of wild type mice and in the brain of AQP11-deficient mice. Cb, cerebellum; Cx, cerebral cortex; Di, diencephalon; Ki, kidney; Th, thymus; Br KO, the whole brain of AQP11-deficient mice.

The expression levels of AQP11 mRNA in various regions of the mouse brain at postnatal 28 days (P28) were examined by real-time reverse transcription (RT)-PCR. The date of P28 was chosen to compare the brain with that of AQP11-deficient mice because most AQP11-deficient mice were dead around one month after birth. AQP11 was similarly expressed in the cerebellum, cerebrum, and diencephalon, but much less than in the kidney (Figure 1A,B). A band (26 kDa) was detected in the brain by immunoblotting, which was absent in the brain of AQP11-deficient mice, a negative control (Figure 1C), confirming the specificity of the antibody. The expression level of AQP11 protein in the brain, however, was also much lower than that in the kidney and thymus (Figure 1C).

### 2.2. Localization of AQP11 in the Brain

**Figure 2:**
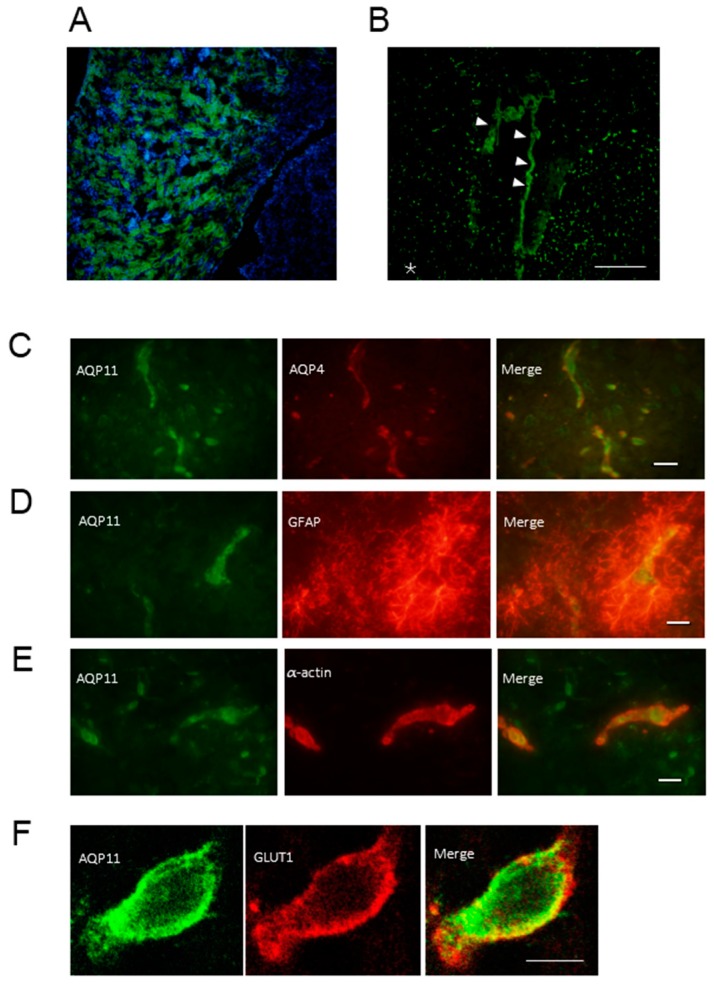
Localization of AQP11 in the kidney and brain (**A**) Immunofluorescence staining for AQP11 (green) and 4′,6-diamidino-2-phenylindole dihydrochloride (blue) for nuclear counterstaining in the kidney of the wild type at P28; (**B**) Immunofluorescence staining for AQP11 (green) in the fourth ventricle of the wild type at P28. The arrow heads indicate the choroid plexus. The asterisk (*) indicates the cerebral grey matter. Scale bar, 200 µm; (**C**–**F**) The double immunofluorescence of AQP11 (green) in the cerebral cortex of the wild type at P28; (**C**) with AQP4 (red; the scale bar, 20 µm); (**D**) with glial fibrillary acidic protein (GFAP) (red; the scale bar, 10 µm); (**E**) with α-actin (red; the scale bar, 10 µm); and (**F**) with GLUT1 (red; scale bar, 5 µm). Sections were viewed and photographed with a fluorescent microscope (**A**–**D**) or confocal scanning microscope (**F**).

The immunohistochemistry of AQP11 showed that AQP11 was expressed in the proximal tubule (Figure 2A) as previously reported [7]. In the brain, AQP11 was expressed abundantly at the choroid plexus but also expressed weakly in the parenchyma (Figure 2B). The specificity of the AQP11 antibody was further confirmed by the absence of the staining in the brain of AQP11-deficient mice (data not shown). In order to examine the detailed localization of AQP11 in the cerebrum, double immunofluorescent staining was employed. The AQP11 staining (green) was not colocalized with the staining of AQP4 (red), a marker for astrocyte endfeet (Figure 2C), glial fibrillary acidic protein (GFAP) (red), a marker for astrocyte (Figure 2D), nor α-actin (red), a marker for pericyte (Figure 2E). Therefore, APQ11 was not expressed at these cells. By contrast, the AQP11 staining (green) partially overlapped with the staining of glucose transporter 1 (GLUT1) (red), a marker for the capillary endothelium (Figure 2F). The result indicated that AQP11 was expressed at the capillary endothelium in the cerebral white matter (Figure 2B). As brain capillary endothelial cells are surrounded by astrocyte endfeet expressing AQP4 and GFAP, AQP11 at the capillary endothelium may function in series with AQP4 at the BBB.

### 2.3. The Expression of AQP11 in the Developing Brain

**Figure 3:**
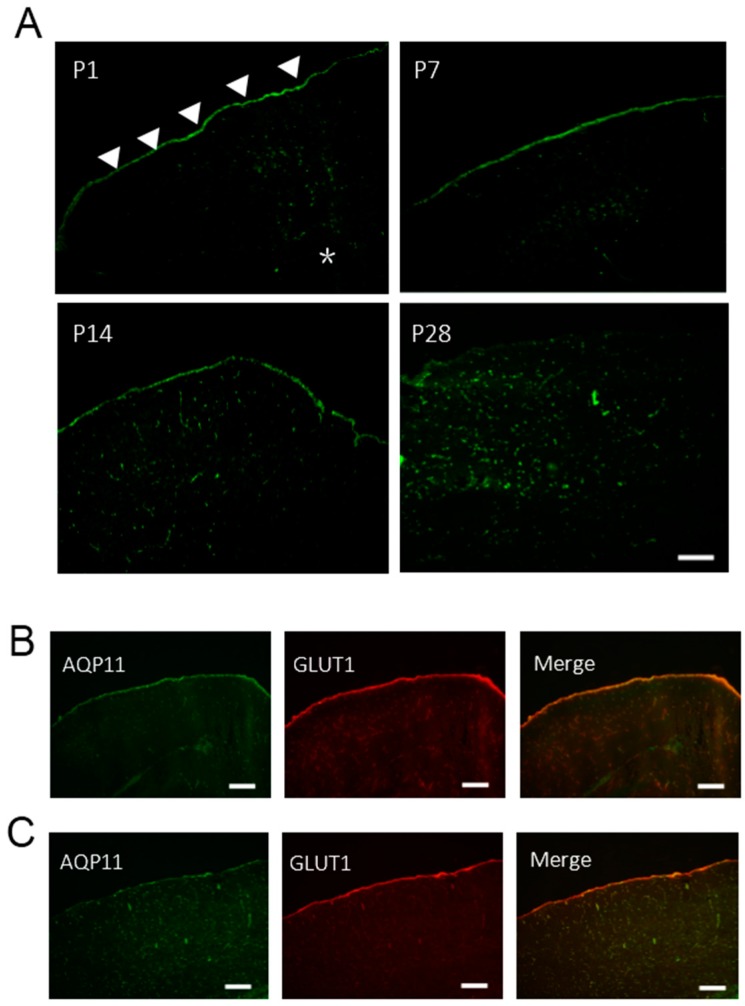
Localization of AQP11 in the brain of P1~P28 (**A**) Immunofluorescence staining for AQP11 (green) in the brain of P1, P7, P14, and P28. Scale bar, 200 µm; (**B**,**C**) Immunofluorescence staining of the cerebral cortex for AQP11 (green) and GLUT1 (red) at P1 (**B**) and P28 (**C**). The arrow heads indicate the pia matter. The asterisk (*) indicates the brain parenchyma. The scale bars, 200 µm.

As neurons were not stained by AQP11 antibodies, we next analyzed the AQP11 expression in the brain at early postnatal stages to observe possible developmental changes of AQP11 expression. AQP11 was mainly expressed at the brain surface with a slight expression in the brain parenchyma of P1, P7, and P14 (Figure 3A,B). With the development of the brain, the intensity of AQP11 expression was shifted from the brain surface to the brain parenchyma. At the stage of P28, AQP11 was mainly expressed in the parenchyma (Figure 3C). Double immunofluorescent staining for AQP11 (green) and GLUT1 (red), a marker for the pia matter and the capillary endothelium, revealed a significant overlap at the brain surface in P1 mouse brain (Figure 3B), while AQP11 was weekly detected at the pia matter but strongly overlapped with GLUT1 in the parenchyma in P28, thereby suggesting the AQP11 expression in the endothelium and not in the neuron and glia (Figure 3C). These results suggest that AQP11 may be important for leptomeninges at the early days after birth but it may become more important for capillary endothelial cells as the brain and the BBB mature. The AQP11 expression in the choroid plexus was constantly observed throughout the brain development from P1 to P28 (data not shown).

### 2.4. The Expression of AQP1 and AQP4 mRNA in the Brain of AQP11-Deficient Mice

**Figure 4:**
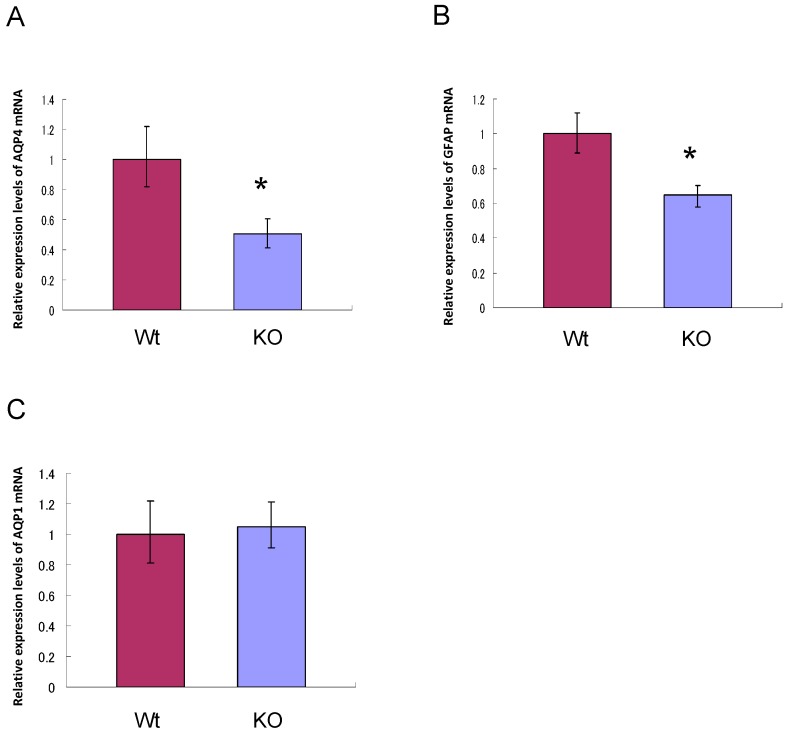
Expression levels of AQP1, AQP4, and GFAP mRNA in the brain of AQP11-deficient mice at P28. (**A**–**C**) Relative abundance of AQP4 (**A**), GFAP (**B**), and AQP1 (**C**) mRNA in the whole brain from AQP11-deficient mice to that in the whole brain of wild-type mice. All signals were normalized to GAPDH mRNA signals. Each bar represents mean ± SD of three experiments. * *p* < 0.05 AQP11-deficient mice *vs.* wild-type mice.

As both AQP1 and AQP4 are also expressed in the brain [16], the expression levels of AQP1 and AQP4 mRNA in the brain were compared between wild-type and AQP11-deficient mice. The mRNA expression levels for AQP4 and GFAP were decreased by half in the brain of AQP11-deficient mice (Figure 4A,B), while that for AQP1 was unchanged (Figure 4C). These results suggest that AQP11 and AQP4 might be functionally coupled in the brain especially at the BBB where both were expressed in series.

### 2.5. The BBB Permeability of AQP11-Deficient Mice Was Normal

**Figure 5:**
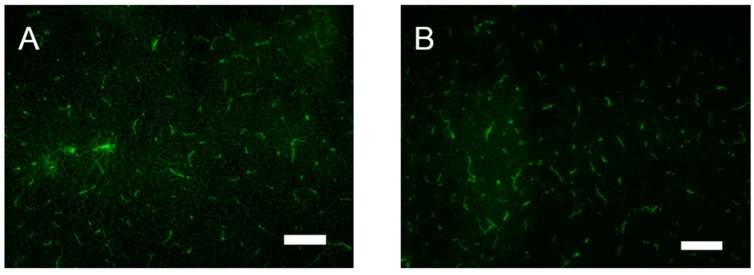
BBB permeability of AQP11-deficient mice was normal. After intracardiac perfusion of sulfo-NHS-biotin, the cerebrum was fixed with 4% paraformaldehyde. Cryosections of frozen tissue were labeled with Alexa-488-streptavidin (green). (**A**) Wild-type mice cerebral cortex; (**B**) AQP11-deficient mice cerebral cortex. Both did not show any leakage. The scale bars, 100 µm.

As both AQP4 and AQP11 were expressed at the BBB, AQP11 null mice may suffer from the BBB dysfunction with concomitant decreased AQP4 expression as well. To evaluate the BBB permeability of AQP11-deficient mice, we stained brain tissue sections for covalent biotin adducts following perfusion of EZ-Link Sulfo-NHS-Biotin reagent (443 D). This reagent has been successfully used to evaluate the permeability of the BBB [17]. As shown in Figure 5, this reagent did not leak into the brain parenchyma in both brains of wild-type and AQP11-deficient mice, indicating that the BBB permeability of AQP11-deficient mice was not compromised. In agreement with the functional data, morphological abnormalities were not observed in the brain of AQP11-deficient mice by light microscopy.

## 3. Discussion

Here we reported for the first time the expression and localization of AQP11 in the mouse brain. Specifically, AQP11 was expressed in the capillary endothelium and the epithelium of the choroid plexus. The reason for the lower expression levels of AQP11 in the brain will be the smaller share of the capillary and choroid plexus in the whole brain which is composed mainly of the neuron and glia unexpressing AQP11.

A previous study, however, has reported that AQP11 has been expressed at Purkinje cell dendrites, hippocampal neurons, and cerebral cortical neurons in the rat brain [16]. The reason for the discrepancy may be the different specificities of the antibodies, as raising antibodies against AQP11 and AQP12 was difficult [18,19]. Our negative blotting in AQP11-deficient mice (Figure 1C) and negative histological staining of the brain of AQP11-deficient mice (data not shown) are supportive for the sufficient specificity of our antibody. On the other hand, the antibody used in the rat study has not stained the kidney [16], thus suggesting a poor sensitivity or specificity of the antibody, as the AQP11 mRNA expression in the kidney is relatively high and its absence produces proximal tubular polycystic kidneys [7].

There were no apparent morphological abnormalities in the brain of AQP11-deficient mice. Although AQP11 is widely expressed, limited abnormalities were observed in AQP11-deficient mice: intracellular vacuoles in the kidney, liver, and intestine and polycystic kidneys [7,20,21]. Similarly, the disruption of other AQPs expressed in the brain, AQP1 or AQP4, has not produced any morphological abnormalities in the brain [13].

AQP1 has been shown to be expressed at the apical surface of the choroid plexus epithelium, whose disruption has decreased the cerebrospinal fluid production by 20%, although no apparent abnormality of ventricles has been observed [14]. It is possible that AQP11 at the epithelium of the choroid plexus may also participate in the production of cerebrospinal fluid. The functional significance of AQP11 in the cerebrospinal fluid production will be a future project.

The AQP11 expression in the brain capillary endothelium was substantiated by double immunofluorescence staining. The AQP11 was colocalized with GLUT1, a capillary endothelial cell marker, but not with GFAP, an astrocyte marker, nor with AQP4, an astrocyte endfoot marker. As AQP11 was not colocalized with α-actin, a pericyte marker, AQP11 is most likely expressed at endothelial cells and not at pericytes. Whether AQP11 is present inside the cell as in the kidney [7] is difficult to conclude for the flat morphology of the endothelium without electron microscopy. Unfortunately, the AQP11 antibody was not specific enough for immune-electron microscopy. As we reported previously [7], AQP11 was not expressed in the capillary of the glomerulus (Figure 2A) nor capillaries in other major organs (data not shown). Thus, the capillary expression of AQP11 seems to be restricted to the brain parenchyma.

The parenchymal capillary in the brain forms the blood-brain-barrier (BBB), which consists of endothelial cells, pericytes, and astrocyte endfeet. The BBB regulates the transport of molecules between the systemic circulation and the brain parenchyma by modulating capillary permeability. AQP4 is expressed at astrocyte endfeet to regulate water transport to the brain parenchyma, as its absence has ameliorated brain edema induced by brain infarction [11] and exacerbated the recovery of brain edema caused by vascular inflammation [12].

Currently, the role of AQP11 in water permeability at the BBB is not clear. As the expression level of AQP4 mRNA was decreased by half in AQP11-deficient mice (Figure 4A), there could be some functional correlations between AQP4 and AQP11, where the absence of AQP11 might downregulate the AQP4 expression at the BBB due to decreased water transport. As the decrease of the AQP4 expression was accompanied by the decrease of the GFAP, abnormal astrocyte development in AQP11-deficient mice brains may also be present. As the permeability of the BBB was normal in AQP11-deficient mice, the integrity of the BBB will be intact. Obviously, it will be necessary to analyze AQP4 expression in distinct brain areas of AQP11-deficient mice since the brain is quite heterogeneous. In fact, we are planning to study brain edema models in AQP11-deficient mice, which may lead to a new therapy against brain edema [22].

Interestingly, AQP11 was expressed mainly in the pia matter with limited expression in the capillary at early postnatal stages at P1~P14. Such a dramatic change of AQP11 expression pattern in the brain from P14 to P28 is intriguing. It is possible that the developing brain may need AQP11 to support the water flow to the growing brain. As the brain development of AQP11-deficient mice seemed to be normal, the role of AQP11 at the pia mater, as well as in the capillary, requires further studies.

## 4. Experimental Section

### 4.1. Animals

All procedures involving animals were approved by the Meiji Pharmaceutical University Committee for Ethics of Experimentation and Animal Care. The generation of AQP11-deficient mice was previous reported [7]. Male C57BL mice at P1~P28 were maintained under a 12 h light/12 h dark cycle with free access to food and water.

### 4.2. RNA Isolation and Quantitative RT-PCR

Total RNAs were isolated from the brain and kidney by RNeasy Lipid Tissue Mini Kit and RNeasy Mini Kit, respectively (Qiagen, Tokyo, Japan), following the manufacturer’s instructions. Each total RNA was reverse-transcribed (RT) by Takara RNA PCR Kit (AMV, version 3.0; Takara, Shiga, Japan) with random 9-mer primers. The expression of AQP11 mRNA in each tissue was examined by RT-PCR using following primers: sense 5′-CTGCTGGCTGCACTCATC-3′ and antisense 5′-TTGAGAAATACAGGCTAC-3′. GAPDH (glyceraldehyde-3-phosphate dehydrogenase) was amplified as an internal control using the following primers: sense 5′-GTGGAAGGACTCATGACCACAGTC-3′ and antisense 5′-TACTCCTTGGAGGCCATGTG-3′. All the primers were from the different exons to suppress the amplification of the genomic clones. PCR protocols for both AQP11 and GAPDH were the following: 30 cycles of (94 °C for 30 s, 58 °C for 30 s, and 72 °C for 1 min). The PCR products were separated in 2% agarose gels.

Quantitative real time RT-PCR was performed using TaqMan^®^RNA-to-CTTM 1-Step Kit (Applied Biosystems, Foster City, CA, USA). RT-PCR amplifications and real-time detection were performed using the ABI Prism 7500 Fast Real-time PCR System (Applied Biosystems). Real-time RT-PCR conditions were the following: 48 °C for 15 min, 95 °C for 10 min, and then followed by 40 cycles of (15 s at 95 °C and 1 min at 60 °C). All purchased TaqMan probes (Applied Biosystems) were from the different exons: GAPDH; Mm99999915_g19, GFAP; Mm01253033_m1, AQP11; Mm00613023_m1, AQP1; Mm00431834_m1, and AQP4; Mm00802131_m1. The results were normalized to GAPDH expression levels. The amount of each gene expression was calculated by using the ΔΔ*C*_t_ method. All experiments were performed in triplicates. Results are shown as a fold change from wild-type mice.

### 4.3. Immunoblotting of AQP11 in the Brain

Brain extracts were prepared from male mice with nine volumes of ice-cold buffer (pH 7.2: 0.3 M sucrose, 25 mM imidazole, 5 mM EDTA, and a complete protease inhibitor cocktail (Roche Diagnostics, Mannheim, Germany)) by using a Potter-type glass homogenizer with a Teflon pestle or a Polytron-type homogenizer. The 80 µg protein extract was incubated for 30 min at room temperature in a sample buffer (2% sodium dodecyl sulfate, 62.5 mM Tris-HCl (pH 6.8), 5% sucrose, 0.001% bromophenol blue, and 125 mM DTT). Each protein was separated by 15% SDS-polyacrylamide gel electrophoresis (PAGE) and then transferred to a polyvinylidene difluoride membrane (GE Healthcare, Buckinghamshire, UK). The blocking of the membrane was performed by ECL Blocking Agent (GE Healthcare). Then, the membrane was incubated for 1 h with a primary antibody against AQP11 [7], followed by three washings with Tris-buffered saline with tween-20 (TBST), and then incubated in a secondary antibody, Anti-Rabbit IgG horse radish peroxidase (HRP)-linked Whole Antibody (GE Healthcare). The protein bands were detected using ECL Plus Western blotting detection reagents (GE Healthcare) in a Typhoon 4910 (GE Healthcare). A relative ratio indicates the relative gene expression levels of AQP11-deficient mice *versus* wild-type mice.

### 4.4. Immunohistochemistry

Mice were anesthetized with diethyl ether and perfused from the heart with phosphate-buffered saline (PBS) for blood removal and then with 4% paraformaldehyde for the fixation. The brain was harvested and then incubated in 4% paraformaldehyde, which was subsequently cryoprotected in a series of sucrose solutions; 10% (4 h), 15% (4 h), and 30% (overnight) sucrose in PBS in succession at 4 °C, and finally embedded in optimal cutting temperature (OCT) compound (Tissue-Tek, Sakura Finetek, Tokyo, Japan) on dry ice with acetone. The brain section was prepared at −20 °C by a cryostat (Leica CM1510S, Leica Microsystems, Heidelberg, Germany), and stored at −80 °C until use. For immunofluorescence staining, the sections were incubated with 0.5% TritonX-100/PBS at 5 min and blocked with 0.1% goat serum in a blocking reagent (0.15% glycine and 0.3% TritonX-100 in PBS) and then incubated with anti-AQP11 antibody (1:500) [7] and with anti-AQP4 antibody (1:100; Santa Cruz Biotechnology, Santa Cruz, CA, USA), anti-α-actin antibody (1:100; Abcam, Cambridge, UK), and anti-glucose transporter-1 (GLUT1) antibody (1:300; Abcam). The immunoreaction was visualized with a secondary antibody (1:1000; goat anti-rabbit Alexa Fluor 488 or goat anti-mouse Alexa Fluor 568; Invitrogen, Tokyo, Japan) at 37 °C for an hour. Sections were viewed with an inverted fluorescent microscope (Olympus IX71, Olympus Optical Co., Ltd., Tokyo, Japan) or a confocal fluorescence Fluoview FV500 microscope (Olympus, Tokyo, Japan).

### 4.5. Biotin Permeability Assay

Biotin permeability assay was performed by following a previously described method with minor modifications [17]. Briefly, deeply anesthetized mice were perfused from the heart with 5 mL 1 mg/mL EZ-Link Sulfo-NHS-Biotin (#21217, Thermo Scientific, Waltham, MA, USA) in PBS, followed by 4% paraformaldehyde in PBS. The tissue was fixed in 4% paraformaldehyde overnight and cryoprotected, embedded in OCT compound, cryosectioned, labeled with Alexa-488-streptavidin, and the images were captured using a fluorescent microscope (Olympus IX71).

### 4.6. Statistical Analysis

Comparisons between two groups were performed using unpaired *t* tests. *p* values <0.05 were considered significant. Data are presented as means ± SDs.

## 5. Conclusions

In conclusion, AQP11 was localized at capillary endothelial cells in the brain parenchyma and epithelial cells of the choroid plexus, but not at neurons and glial cells. However, no apparent morphological abnormalities were observed in the brain of AQP11-deficient mice. Its restricted localization at the BBB and the down-regulation of AQP4 expression in AQP11-deficient brains suggest that AQP11 may play a role in the BBB water transport and in the pathophysiology of brain edema.
